# Molecular mechanism of colorectal cancer and screening of molecular markers based on bioinformatics analysis

**DOI:** 10.1515/biol-2022-0687

**Published:** 2023-11-10

**Authors:** Jikun Zhao, Dadong Kuang, Xianshuo Cheng, Jiwei Geng, Yong Huang, Haojie Zhao, Zhibin Yang

**Affiliations:** Department of General Surgery, Baoshan People’s Hospital in Yunnan Province, Baoshan 678000, China; Department of Colorectal Surgery, Yunnan Cancer Hospital, Kunming 650118, China; Department of Oncology, Baoshan People’s Hospital in Yunnan Province, Baoshan 678000, China

**Keywords:** bioinformatics, colorectal cancer, pathogenesis, molecular marker screening

## Abstract

Genomics and bioinformatics methods were used to screen genes and molecular markers correlated with colorectal cancer incidence and progression, and their biological functions were analyzed. Differentially expressed genes were obtained using the GEO2R program following colorectal cancer chip data GSE44076 retrieval from the Gene Expression Omnibus gene expression comprehensive database. An online database (David) that combines annotation, visualization, and gene discovery was utilized for investigating genes. Pathway and protein analyses were performed via resources from the Gene Ontology (GO) and the Kyoto Encyclopedia of Genes and Genomes (KEGG). Visual analysis of the KEGG pathway was carried out according to ClueGO and CluePedia to establish the PPI network of gene interaction between pathways; the genes with the highest connectivity were screened by the molecular complex detection analysis method as Hub genes in this study; gene expression was verified by GEPIA online analysis tool, and Kaplan–Meier survival curve was drawn for prognosis analysis. By analyzing GSE44076 microarray data, 86 genes were selected, and colorectal cancer tissues’ upregulation was observed in 27 genes and downregulation in 59 ones. GO assessment revealed that the differentially expressed genes were basically correlated with retinol dehydrogenase activity, carbon dehydrogenase activity, collagen-containing extracellular matrix, anchored component of memory, and cellular hormone metabolic process. Moreover, the KEGG assessment revealed that the differential genes contained various signal pathways such as retinol metabolism, chemical carotenogenesis, and nitrogen metabolism. Through further analysis of the PPI protein network, 4 clusters were obtained, and 16 Hub genes were screened out by combining the degree of each gene. Through the analysis of each gene on the prognosis of colon cancer through the GEPIA online analysis website, it was found that the expression levels of AQP8, CXCL8, and ZG16 genes were remarkably associated with colon cancer prognosis (*P* < 0.05). Genomics and bioinformatics methods can effectively analyze the genes and molecular markers correlated with colorectal cancer incidence and progression, help to systematically clarify the molecular mechanism of 16 key genes in colorectal cancer development and progression, and provide a theoretically valid insight for the screening of diagnostic markers of colorectal cancer and the selection of accurate targets for drug therapy.

## 1 Introduction

Colon cancer has elevated incidence and fatality rates and is a leading etiology of cancer-associated morbidity globally [1]. Clinically, colon cancer treatment mainly adopts the comprehensive treatment of surgical resection and postoperative chemotherapy according to the condition, which has achieved certain results. However, because most patients in the middle and advanced stages have developed distant metastasis, the treatment effect is not good, and the mortality rate is still high [2]. Early diagnosis of colon cancer and early treatment can be vital to diminish impacted patients’ mortalities. Colon cancer pathogenesis is not clear, and it is believed that it may be regulated by many factors and genes [3]. Hence, in the context of colon cancer, the pathological characteristics are not readily apparent. Therefore, it is crucial to conduct screening and analyze the abnormal expression profile of genes associated with colon cancer. This analysis aims to construct an interaction network of abnormal genes and identify diagnostic markers. Additionally, the identification of Hub genes with high connectivity in the network is essential for understanding the underlying mechanisms of the disease and ultimately reducing mortality rates [4]. Some studies have proposed that [5], abnormal gene expression caused by epigenetic changes is one of the important factors in cancer occurrence. Since the advent of modern technological advancements, people gradually analyzed various disease pathogeneses from the gene level, and the human gene data are huge, and the high throughput of sequencing technology provides a convenient and in-depth analysis method for many genetic data [6]. In this study, GSE44076 of colon cancer microarray data from gene expression comprehensive database (Gene Expression Omnibus, GEO) database as the experimental object, screening the differential expressed genes in colon cancer tissue and normal tissue, functional enrichment analysis, screening genes remarkably correlated with colon cancer molecular mechanisms, and Kaplan–Meier survival curve, to provide valuable molecular markers for early prevention, accurate diagnosis and treatment of colon cancer patients.

## 2 Materials and methods

### 2.1 Experimental subject

The colorectal cancer microarray data GSE44076 was downloaded from GEO. This dataset obtained colon adenocarcinoma pathological tissues and distal normal colon mucosa tissues from 98 cases, as well as gene expression profiles of 50 healthy human colon mucosa through Affymetrix human genome U219 array. Research on this dataset came from the colonomics project (www.coloonomics.org). In the present study, the colon adenocarcinoma tissues from 98 patients were included in the observation group (total 98 samples), and the normal mucosa distal to colon adenocarcinoma with healthy human colon mucosa was included in the control group (total 148 samples).


**Informed consent:** Informed consent has been obtained from all individuals included in this study.
**Ethical approval:** The research related to human use has been complied with all the relevant national regulations, institutional policies and in accordance with the tenets of the Helsinki Declaration, and has been approved by the authors' institutional review board or equivalent committee.

### 2.2 Cell culture

Three cell lines were used in this study, namely human colon adenocarcinoma cells (SW48), human normal colonic epithelial cells (NCM460), and human colon carcinoma cells (HT29). Cells were grown in 100 U/mL penicillin medium, 37℃, 100 mg/mL streptomycin, and 10% fetal bovine serum (FBS) (10% penicillin–streptomycin solution together with 10% FBS and 90% high glucose Dulbecco’s modified eagle medium). Cells were passaged when the cultures reached 80% confluence.

### 2.3 Immunocytochemistry

After fixing NCM460, SW48, and HT29 cells in 4% paraformaldehyde solution for 15 min at room temperature, washing them three times in phosphate-buffered saline (PBS), and blocking them with 10% goat serum, slides were produced. The samples were incubated with rabbit anti-human AQP8 (ab133667, 1:1500) and ZG16 (ab185483, 1:1500) antibodies for one more night at 4℃. Vectashield (Vector Laboratories Inc., Burlingame, CA) was utilized for sealing the samples after they were washed three times with PBS and treated with goat anti-mouse FITC/APC conjugated IgG H & L secondary antibody for 45 min. The cells on the slides were seen and recorded on film via a forward fluorescent microscope (Olympus, Tokyo, Japan).

### 2.4 Reverse transcription-quantitative polymerase chain reaction (RT-qPCR)

Tissues and cells were treated with TRIzol (15596026, Invitrogen) for removing their total RNA. The RT kit (RR047A, Takara, Japan) could be utilized for converting RNA to complementary DNA, and then, the samples were mixed with the SYBR Premix EX Taq kit (RR420A, Takara). A real-time fluorescence qPCR device (ABI7500; ABI; Foster City, CA, USA) was used to conduct the RT-qPCR. Each sample was placed in its own set of three wells. Sangon Biotech Co., Ltd. (Shanghai, China) produced all primers (Table S1). Internal standard glyceraldehyde-3-phosphate dehydrogenase (GAPDH) Ct values were recorded. Utilizing 2^−ΔΔCt^, a relative quantitative method, we determined the fold change between the experimental and control groups.

### 2.5 Western bloting

The protein levels of AQP8 and ZG16 were detected. To begin, samples were washed with PBS before being homogenized in RIPA lysis buffer. BCA (Beyotime, P0010) could be utilized for measuring protein concentrations. Sodium dodecyl sulfate-polyacrylamide gel electrophoresis could be carried out with equal quantities of loaded protein. After transferring proteins to a PVDF membrane, they were incubated overnight at 4°C with rabbit anti-human antibody (ab133667, 1:1,500), anti-ZG 16 rabbit anti-human antibody (ab185483, 1:1,500), and β-actin (ab8226, 1:1,500), with β-actin serving as an internal standard. After priming with the primary antibody (ab97051, 1:2,000), we employed a goat anti-rabbit secondary antibody that was coupled with horseradish peroxidase. Quantity One v4.6.2 was utilized for the analysis of images collected via the Bio-Rad picture analysis system (Bio-Rad, CA, USA). The ratio of the gray value of the target band to the gray value of the β-actin band is a measure of relative protein expression. Three sets of trials were performed, and the average gray values were calculated.

### 2.6 Experimental tools

The National Center for Biotechnology Information (NCBI) is primarily responsible for developing and maintaining GEO, the world’s biggest and most comprehensive worldwide public data resource platform. At present, the GEO database has stored several data sets, a series of platforms, samples, and so on.

### 2.7 Differential gene analysis

The GEO2R [4] platform is an interactive analysis tool for the R language-based analysis of differential expressions between group 2 and above in the GEO database. Data Series Matrix File (s) of colon cancer chip from GEO database, analyzed data of colon cancer tissues and normal tissues by GEO2R platform, obtained differentially expressed genes, data were screened by gene fold change (Fold change, FC), |log(FC)| > 3, and *P* < 0.05 was selected as the inclusion condition.

### 2.8 Gene ontology (GO) functional enrichment analysis

GO database analysis is an important bioinformatics tool developed by the Gene Ontology Alliance, which can explain the three structured networks of cell composition, analysis function, and biological process. The R software package ClusterProfiler was utilized to explore relationships among differentially expressed genes. A GO enrichment analysis was conducted using the online database DAVID, known for its comprehensive annotation, visualization, and gene discovery features. This facilitated a focused investigation into GO term enrichment within the gene set. *P* < 0.05 was selected as the inclusion condition.

### 2.9 Kyoto encyclopedia of genes and genomes (KEGG) pathway enrichment analysis

KEGG analysis can be utilized as a biological system database on genomes, enzymatic pathways, and biochemicals established by Japanese scientists in 1995. KEGG pathway enrichment analysis was deployed via the language R software package clusterProfiler, which can reveal the biological pathways of some disease-related genes and drugs. *P* < 0.05 was selected as the inclusion condition.

### 2.10 Protein–protein interaction (PPI) network construction

PPI network can identify the core differentially expressed genes and key gene modules between healthy individuals and patients. The protein interaction pairs were conducted using the online database analysis software STRING. Subsequently, the Cytoscape software platform was utilized to develop the Gene PPI network. The genes were ranked based on their connectivity within this network, and the more aggregated expression subset was selected to discover potential regulatory genes for colon cancer. After that, KEGG pathway visualization analysis was conducted using ClueGO and CluePedia. This analysis facilitated the establishment of a gene-interaction network connecting pathways. Subsequently, a comprehensive screening was conducted to identify differentially expressed genes that had substantial associations with these pivotal pathways.

### 2.11 Colorectal cancer Hub gene screening

Hub genes are a group of genes with the highest connectivity at the center of the network. Genes with more than 10 connections are often used as Hub genes. Molecular complex detection (MCODE) is a strategy for detecting tightly connected PPI network regions. In this study, high-connectivity genes in the PPI network were identified using MCODE analysis as potential Hub genes.

### 2.12 Differential expression analysis validation

The screened differential gene expression in colon cancer sample tissues was viewed through the GEPIA online database, and Kaplan–Meier survival curves were plotted for the genes that were differentially expressed for prognostic analysis.

### 2.13 Statistical methods

Various information data screened were statistically analyzed using various data analysis software introduced in the above methods.

## 3 Results

### 3.1 Screening of genes differential expression in colon cancer and normal tissues

**Figure 1:**
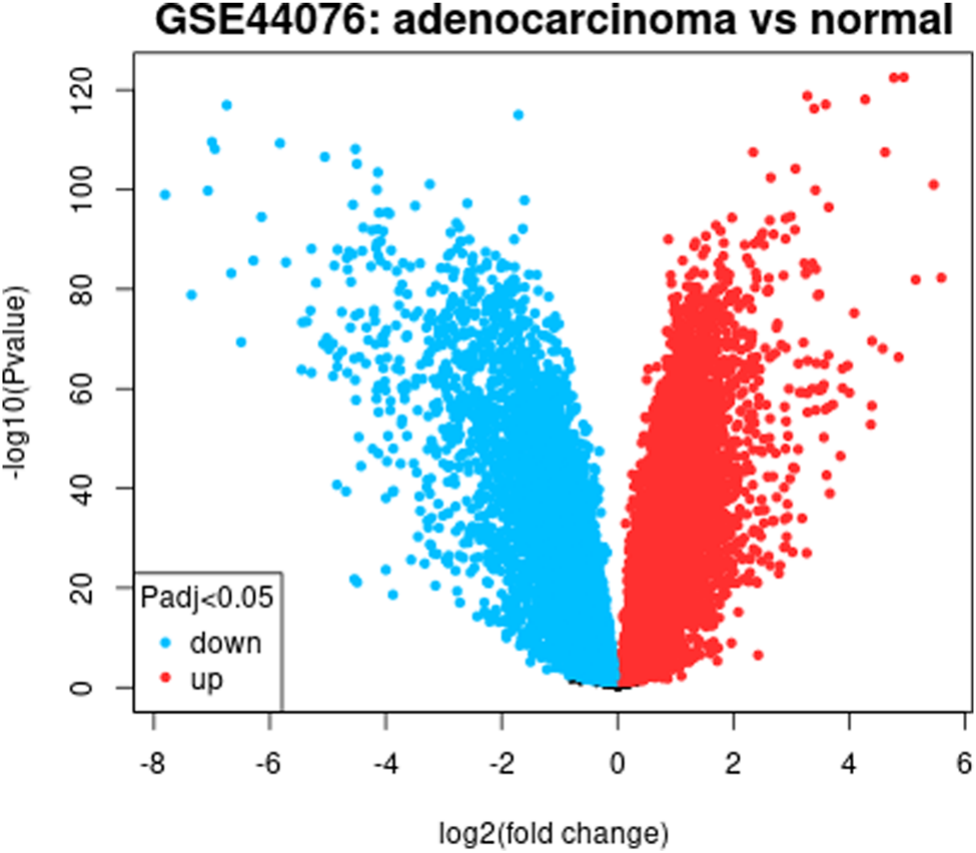
Volcano plots of up- and downregulated genes in the GSE44076 dataset.

GEO2R analysis showed that 86 genes with differential expression of |log(FC)| > 3 were selected from GSE44076 chip data, and colorectal cancer tissue upregulation was observed in 27 genes, and downregulation in 59 ones. The volcano map of related genes is shown in Figure 1.

### 3.2 KEGG pathway and GO functional enrichment and analyses

**Figure 2:**
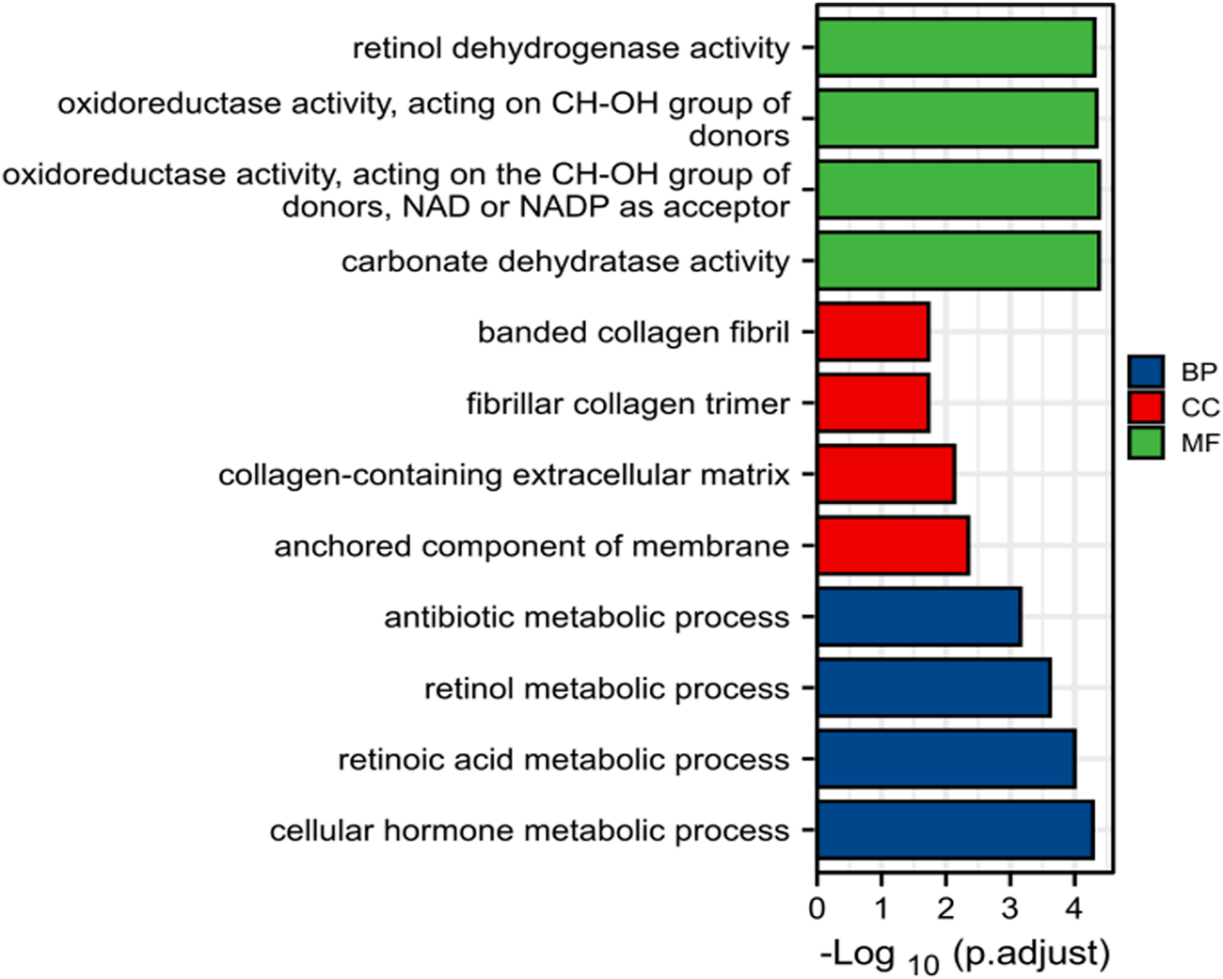
GO analysis of the differential genes showed MF, CC as well as BP.

The differentially expressed genes data were imported into the David online network bioinformatics analysis website (www.david.org) and the findings from the GO assessment revealed that for the genes with differences in biological processes on molecular function (MF), mainly involved in retinol dehydrogenase activity, carbonate dehydrogenase activity, etc.; on the cell component (CC) is mainly involved in collagen-containing extracellular matrix, anchoring component of membrane; In biological process (BP) mainly involved in processes such as cellular hormone metabolic process, see Figure 2.

**Figure 3:**
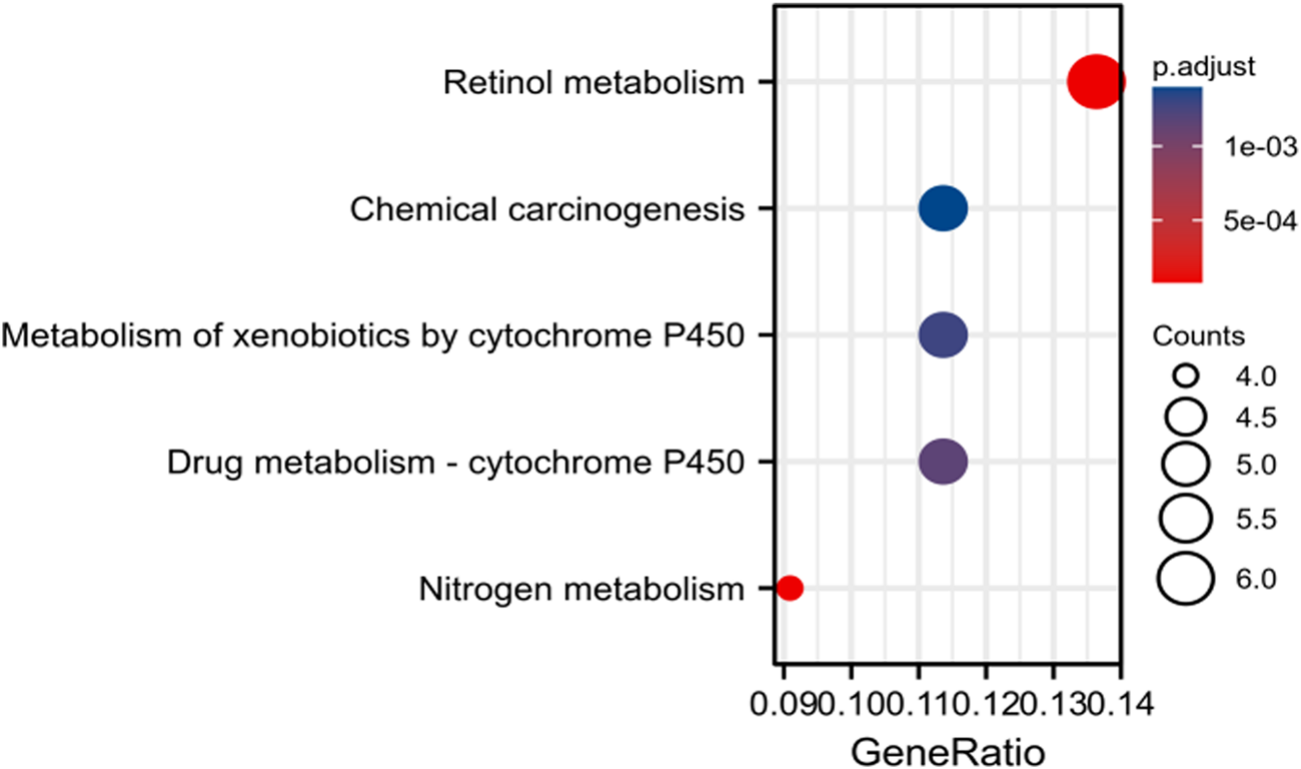
KEGG analysis of differential genes revealed genes mainly enriched signaling pathways.

KEGG signaling pathway analysis revealed that the differential genes were enriched in retinol metabolism, chemical carcinogenesis, nitrogen metabolism, etc., signal channel, see Figure 3.

### 3.3 PPI network construction and screening and identification of Hub genes

**Figure 4:**
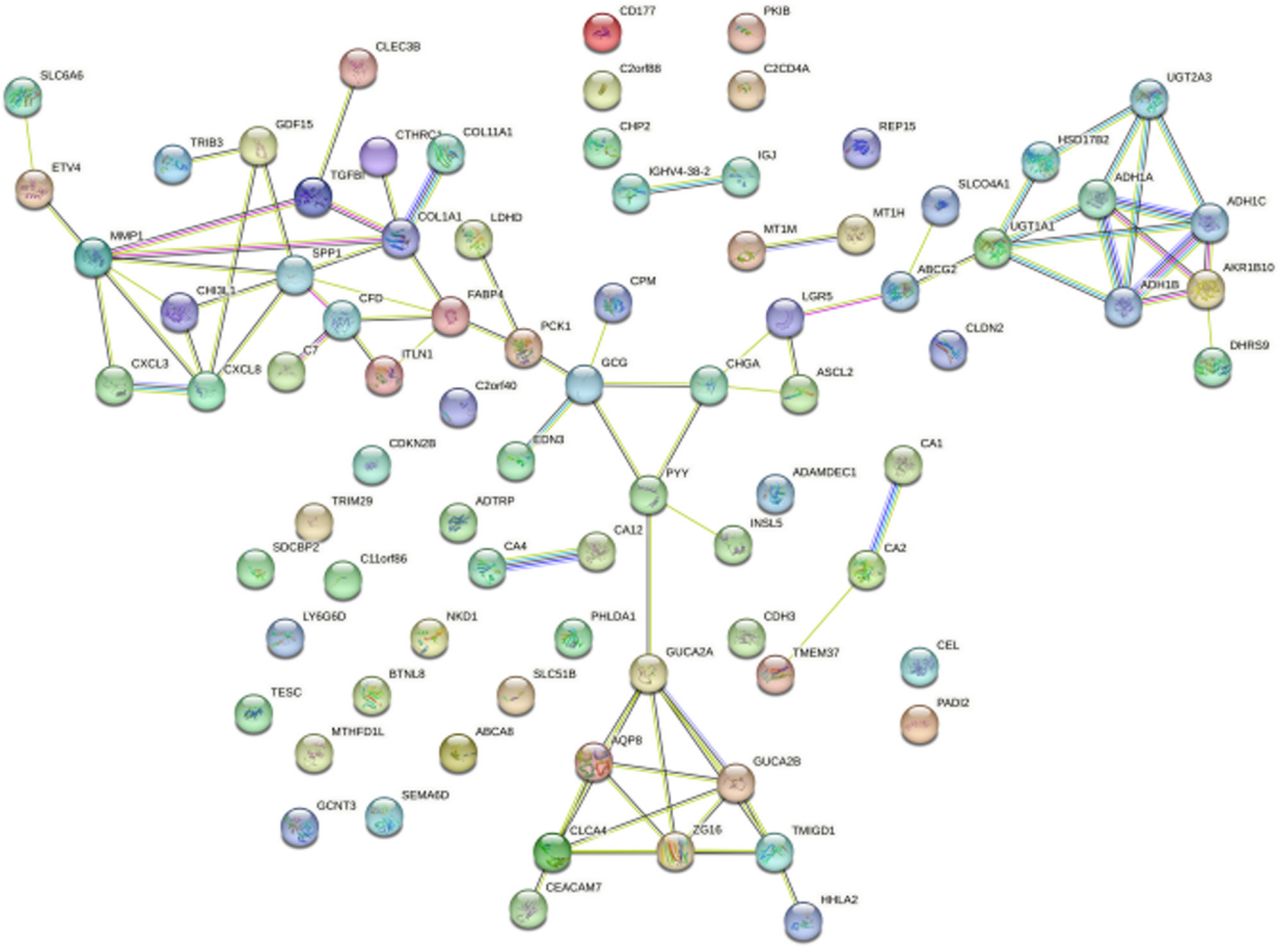
The PPI network constructed by the differentially expressed genes.

The filtered differentially expressed genes were entered into the online data analysis software STRING website to construct the PPI network, see Figure 4.

### 3.4 MCODE analysis

**Figure 5:**
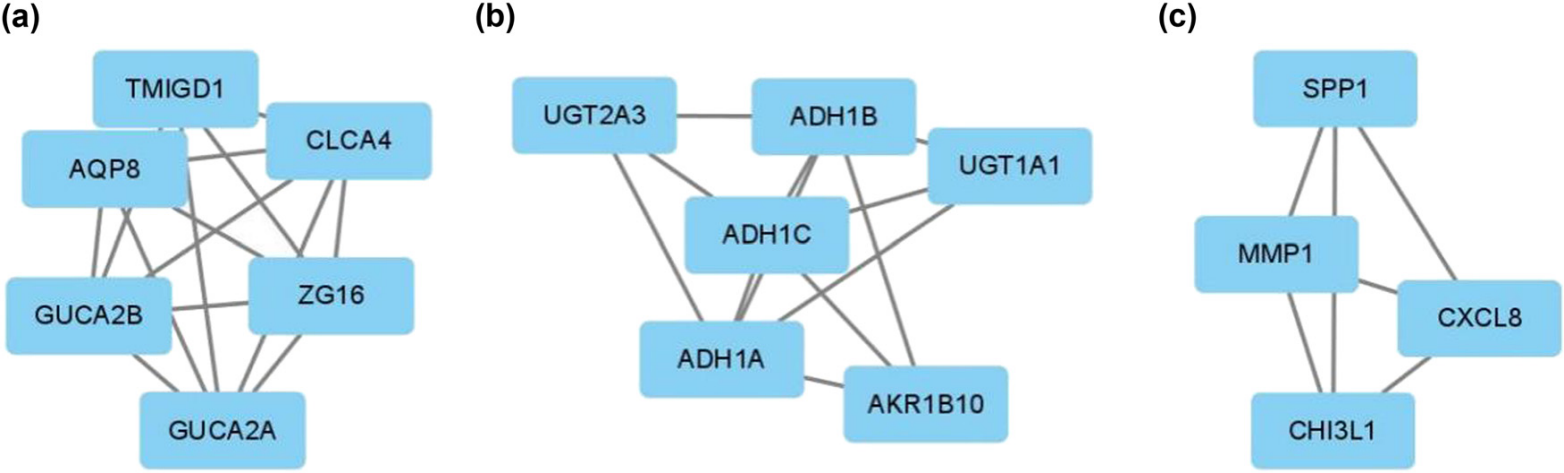
The PPI network analysis using MCODE in Cytoscape software identified three modules corresponding to differentially expressed genes (labeled a-c). These three modules consist of 16 genes with significant connectivity and were subsequently screened for Hub genes associated with colon cancer.

MCODE analysis was used to group the most closely connected genes in the PPI network and showed that a total of three meaningful modules were divided in the total PPI network. The three modules contained 16 genes with high connectivity and were screened for Hub genes in colon cancer, see Table 1 and Figure 5.

**Table 1:** The three modules with higher connectivity and their included genes in the PPI network constituted by differentially expressed genes

Clusters	MCODE score	Degree	Name
Cluster 1	3.7	6	CLCA4
	3.7	6	GUCA2A
	3.7	5	GUCA2B
	3.7	5	ZG16
	4	5	TMIGD1
	4	4	AQP8
Cluster 2	3	5	UGT1A1
	2.4	5	ADH1A
	2.4	5	ADH1C
	2.4	5	ADH1B
	3	4	AKR1B10
	3	4	UGT2A3
Cluster 3	3	7	SPP1
	3	7	MMP1
	3	5	CXCL8
	3	3	CHI3L1

### 3.5 GEPIA online database validation

**Figure 6:**
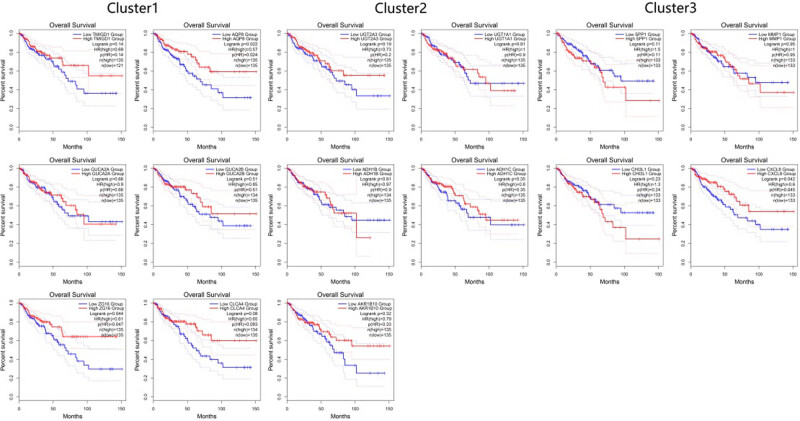
The relationship between the Hub gene and the prognosis of colon cancer patients was analyzed by the GEPIA online tool.

Survival analysis of the screened Hub genes was performed using the online analysis tool GEPIA. The analysis results showed that the expression levels of AQP8 (*P* = 0.022), CXCL-8 (*P* = 0.042), and ZG16 (*P* = 0.044) were significantly associated with the prognosis of colon cancer, and the high expression of the above three genes was related to their good prognosis, see Figure 6.

### 3.6 AQP8 and ZG16 showed low expression in colon cancer cells

**Figure 7:**
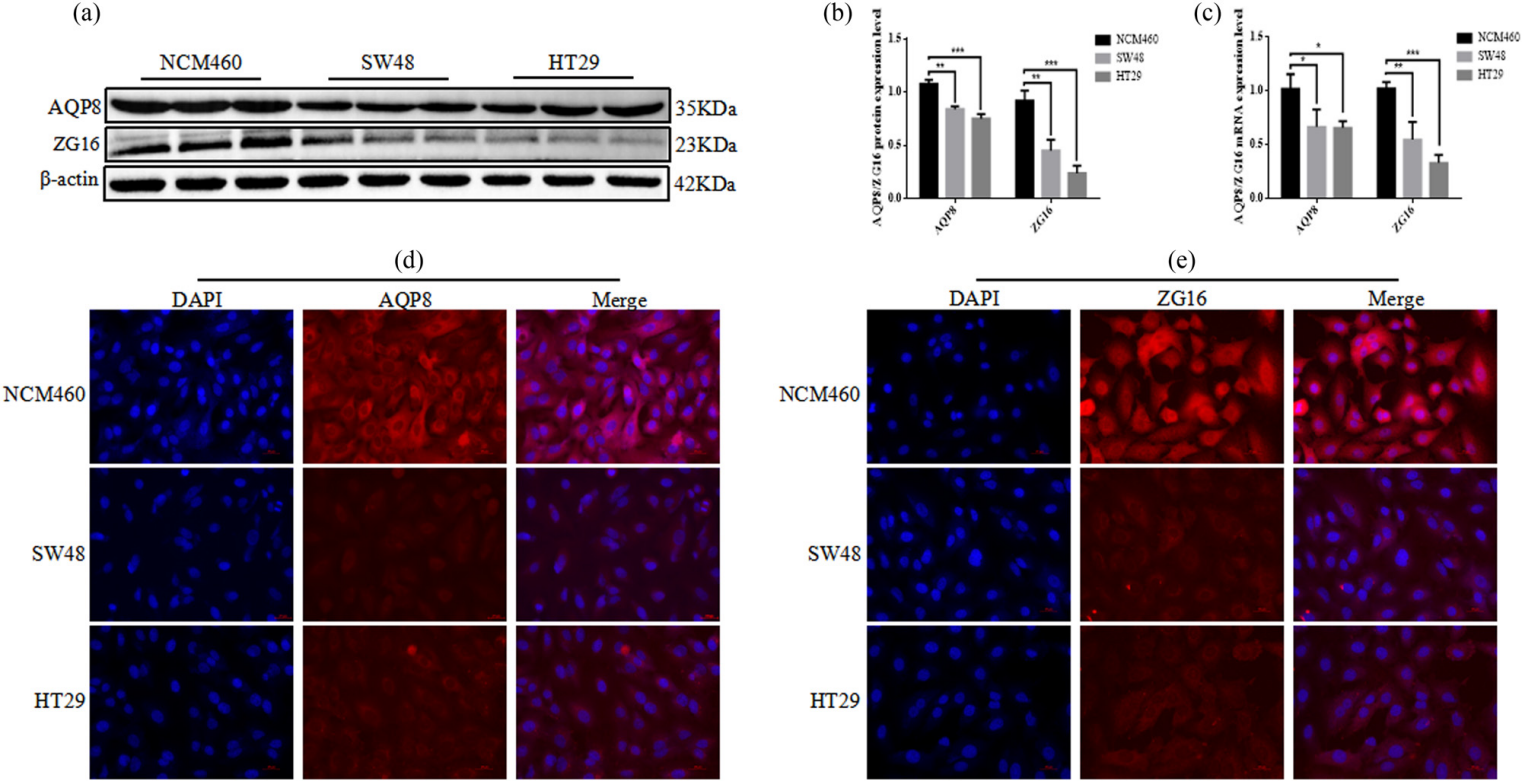
AQP8 and ZG16 in colon cancer cells. (a) and (b) The protein expression levels of AQP8 and ZG16 were determined by Western blotting. (c) The mRNA expression levels of AQP8 and ZG16 were determined by qRT-PCR. (d) and (e) Cellular immunohistochemical staining.

The expression levels of the two Hub genes, AQP8 and ZG16, whose expression levels were significantly associated with the prognosis of colon cancer, were examined in human normal epithelial cells (NCM460) and colon cancer cells (SW48 and HT29). Western blotting analysis detected significantly lower expression levels of AQP8 and ZG16 in SW48 and HT29 cells than in NCM460 cells (Figures 7a and b). Subsequently, qRT-PCR analysis showed that AQP8 and ZG16 expression levels were also significantly lower in SW48 and HT29 cells than in NCM460 cells (Figure 7c). Cell immunohistochemical staining results also showed that the expression levels of AQP8 and ZG16 in SW48 and HT29 cells were also significantly lower than those in NCM460 cells (Figure 7d and e).

## 4 Discussion

Clinically, colorectal cancer is among the most frequent malignant tumors of the digestive system. The rising risk of colorectal cancer is directly linked to the rising level of living and the subsequent shift in individual lifestyles. It is now the fourth cancer in the world, second only to lung cancer, liver cancer, and stomach cancer, which seriously affects people’s quality of life [7]. With poor clinical outcomes in patients at the intermediate and advanced stages, the standard clinical treatment for colon cancer nowadays is surgical resection coupled with radiation and chemotherapy. The fast advancement of molecular biology tools has greatly improved the detection and management of colon cancer, but the pathophysiology is still poorly understood. More and more research suggests that numerous genes and signaling pathways may be involved in the initiation and progression of cancers [8,9].

Bioinformatics is an interdisciplinary subject developed with the human genome project, including database analysis, sequence alignment analysis, gene chip data analysis, biomolecular network analysis, gene regulation network research, and many other aspects, which influences the initiation and progression of early diagnosis of illnesses. Since the advent of modern technological advancements, modern efficacious sequencing technologies and gene chip technology are gradually developed, and cancer research is one of the most widely used fields in disease research [10]. Some studies have found that [11], screening for genes associated with chemotherapeutic neutropenia in breast cancer patients through genome-wide association sequencing data, plays an important role in preventing death due to neutrophil reduction in patients after chemotherapy. The gene expression profile microarray provides an important role in analyzing differential genes between tumor and normal tissue and searching for new tumor markers, which for screening gene markers for tumor classification and classification as well as potential drug therapy targets.

This research used the publicly available microarray data on colon cancer (GSE44076) as its object, performing KEGG pathway and GO functional enrichment analyses on the chosen differential genes. These analyses revealed that the differential genes were primarily involved in processes involving retinol dehydrogenase activity, carbonate dehydratase activity, collagen-containing extracellular matrix, anchored component of membrane, and cellular hormone metabolism. Signaling pathways such as retinol metabolism, chemical carcinogenesis, and nitrogen metabolism were shown to be enriched in genes that were differentially expressed. The above-mentioned biological mechanisms and signaling pathways have all been linked to the emergence and progression of colon cancer [12,13,14,15].

The most closely connected genes in the PPI network were grouped by MCODE analysis method. The three modules with higher scores included 16 genes, such as ZG16, AQP8, UGT2A1, UGT2A3, CHI3L1, CXCL8, with high connectivity, and were screened as Hub genes. Among them, according to GEPIA database analysis, AQP8, CXCL8 and ZG16 are significantly linked to colon cancer patients’ prognosis, and the low expression of the above three genes is associated with poor prognosis.

AQP8 (aquaporin 8) is widely expressed on the cell membrane, mainly involved [16] in physiological and pathological processes such as gland secretion, pulmonary fluid transport, intestinal fat digestion and absorption, and cerebral edema formation. Different investigations concluded that AQP8 diminished expression is remarkably linked to the development of colorectal cancer, cervical cancer, and pancreatic ductal cancer [17]. In this study, AQP8 was considered as the Hub gene in the differential genes of colon cancer and control tissues, and in the GEPIA online analysis showed that AQP8 expression was clearly associated with colon cancer prognosis, and patients having low AQP8 expression had poor prognosis of patients with higher expression.

CXCL8 belongs to the CXC chemokine family, which is secreted by macrophages, epithelial cells, and other cells. It is considered to be a potent chemotactic stimulant. It is related to [18] and immune resistance induced by tumor-associated neutrophils (TANS) and epithelial–mesenchymal transition. G-protein-coupled receptor (GPCR), CXCR1, and CXCR2 are examples of cell surface receptors that mediate CXCL8’s primary activities. In addition, CXCL8 has different biological roles [19] when interacting with different receptors. For example, when binding to CXCR1, it mainly promotes tumor cell proliferation and can promote angiogenic [20] in tumor tissues. It has been suggested that patients with colon adenocarcinoma with high CXCL8 expression have a worse prognosis [21]. However, in this study, cases whose CXCL8 expression levels were low were shown to have a worse prognosis in the GEPIA database assessment. Selection bias may be responsible for this discrepancy, as we have found.

Epithelial cells of the digestive tract are particularly rich in expression of ZG16 (zymogen granule protein 16), which plays an anti-inflammatory, antitumor purpose by disassociating bacteria from the colonic epithelium to prevent local inflammation [22]. The current study shows that ZG16 is generally underexpressed in colorectal cancer tissues and that patients with lower expression levels of ZG16 in colorectal cancer tissues have a worse prognosis [23]. This agrees with the current study’s findings and shows that ZG16 may be employed as a prognostic indicator in individuals with colorectal cancer.

In conclusion, CLCA4, GUCA2A, and GUCA2B were detected by GSE44076 screening of colon cancer microarray data, ZG16, TMIGD1, AQP8, UGT1A1, ADH1A, ADH1C, ADH1B, AKR1B10, UGT2A3, SPP-1, MMP-1, CXCL-8, CHI3L1, and other 16 genes have abnormal expression in multiple tumors, the 16 genes are located in the key node of colon cancer expression network composed of differentially expressed genes, can be regarded as key genes in the development of colon cancer, and by GEPIA online database analysis shows that AQP8, CXCL-8, and ZG16 have a significant correlation with colon cancer patients’ prognosis, providing a theoretically valid importance for further study of the molecular mechanism and molecular marker screening of colon cancer.

## Supplementary Material

Supplementary Table
