# Supplementary material for: Molecular mechanism of colorectal cancer and screening of molecular markers based on bioinformatics analysis
Source: Open Life Sci. 2023 Nov 10;18(1):20220687. doi: 10.1515/biol-2022-0687 (PMC10638842; doi:10.1515/biol-2022-0687)
Supplement: Supplementary Table [file biol-2022-0687-sm.pdf]

Supplementary material

Table S1: The primer sequence

Gene name		Sequence （5′–3′）
AQP8	Forward primer	GTGCCTGTCGGTCATTGAGA
	Reverse primer	CAGTACGGGAGGAGCATCAC
ZG16	Forward primer	TGGCACAAGTTTCAATGCCG
	Reverse primer	GCTGCAGCTACTGGGGTAAA
GAPDH	Forward primer	GGGCTCTCCAGAACATCATCC
	Reverse primer	GGTCCACCACTGACACGTTG
